# Sustained impact of bivalent HPV immunisation on CIN incidence over two rounds of cervical screening

**DOI:** 10.1002/ijc.70183

**Published:** 2025-11-03

**Authors:** Timothy J. Palmer, Kimberley Kavanagh, Kate Cuschieri, Ross L. Cameron, Catriona Graham, Allan Wilson, Kirsty Roy

**Affiliations:** ^1^ Public Health Scotland Glasgow UK; ^2^ MRC Centre for Reproductive Health University of Edinburgh Edinburgh UK; ^3^ Department of Mathematics and Statistics University of Strathclyde Glasgow UK; ^4^ Scottish HPV Reference Laboratory Royal Infirmary of Edinburgh Edinburgh UK; ^5^ Edinburgh Clinical Research Facility University of Edinburgh, Western General Hospital Edinburgh UK; ^6^ Scottish Cervical Screening Programme, National Services Division (NSD) Edinburgh UK

**Keywords:** age at immunisation, bivalent HPV vaccination, herd protection, high‐grade CIN, socio‐economic deprivation

## Abstract

Vaccination against high‐risk HPV has been shown to reduce significantly the incidence of pre‐invasive and invasive cervical disease. Clinical trials show immunity and vaccine effectiveness for over 12 years but real‐life longitudinal data are lacking. Vaccination with the bivalent vaccine (3 dose schedule) for women aged 12–18 years began in Scotland in 2008; immunised women entered screening in 2010. Women immunised at age12–13 years entered screening in 2015. Linked data from ≤12 years of routine screening activity shows adjusted VE against CIN2+ at age 12–13 of 72·6% (95%CI: 67.7–76.8) and at age 14–16 of 63·2 (CI 60·4–65·8), and against CIN3+ of 81·7 (CI 76·2–78·6; age 12–13) and 68·1 (CI 64·1–71·6; age 14–16) after 3 doses or two doses 5 months apart. Adjusted VE following two doses 1 month apart or one dose only at age14 was 20.5 (95%CI: 6.1–32.6) for CIN2+ and 34.9 (95%CI:17.0–48.9) for CIN3+. No benefit was seen with vaccination over the age of 18 years. The most deprived women showed the highest incidence of CIN2+ and CIN3+, and the greatest reduction in CIN2+ and CIN3+ following complete immunisation. Herd protection is seen in all immunised cohorts. This population based analysis confirms the long‐term effectiveness of the bivalent HPV vaccine, greatest in women from the most deprived areas and reinforces the importance of ensuring high vaccine uptake rates at an early age.

## What's New?

While vaccination against high‐risk HPV infection, the primary cause of cervical malignancy, has demonstrated long‐term effectiveness in clinical trials, real‐life longitudinal data are lacking. Here, the authors present linked long‐term screening data on the effectiveness of bivalent HPV vaccination with a minimum follow‐up of 54 months. The results show high vaccine effectiveness against high‐grade cervical intraepithelial neoplasia in women who were vaccinated aged 12–13 years, significant effectiveness in women vaccinated aged 14–16 and 17–18 years with herd protection, and no benefit in women vaccinated over 18. The findings confirm stable protection from bivalent HPV vaccination.

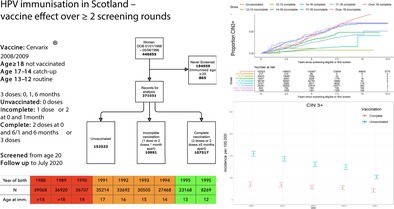

**Abbreviations**
CHI: community health index
CIN: cervical intraepithelial neoplasia
CoxPH: Cox proportional hazard
GBMSM: gay, bisexual and other men who have sex with men
HR: hazard ratio
hr‐HPV: high‐risk Human Papillomavirus
ICD: International Classification of Diseases
JCVI: Joint Committee on Vaccines and Immunisation
LMIC: low and middle‐income countries
py: person‐years
SCCRS: Scottish Cervical Call‐Recall System
SIMD: Scottish Index of Multiple Deprivation
SNOMED: Systematised Nomenclature of Medicine
VE: vaccine effectiveness

## 1 INTRODUCTION

Cervical cancer is recognised as a significant world‐wide public health problem and is associated with considerable mortality and morbidity, particularly in low and middle income (LMIC) settings.^1^ In high income countries considerable resources, not available in LMIC, are devoted to screening programmes to limit the extent of the disease. In 2018, the WHO called for the elimination of cervical cancer as a public health problem in the world, recognising screening, treatment and high‐risk Human Papillomavirus infection (hr‐HPV) vaccination would each have fundamental roles to play. Hr‐HPV infection is the primary cause of most pre‐invasive and invasive cervical malignancy, and the current vaccines have demonstrated long‐lasting immunity, preventing infection by the target HPV and also pre‐invasive disease over periods up to 14 years.^2^, ^3^ Whilst early population studies and systematic reviews show encouraging results,^4^, ^5^, ^6^ questions still remain concerning the long term clinical efficacy of the vaccines, HPV type replacement in cervical intraepithelial neoplasia (CIN), ‘unmasking’ of non‐vaccine types in multiple infections, and the appropriate responses of screening programmes and colposcopy to the falling rates of CIN.

Scotland has had an organised cervical cancer screening programme since 1990 and introduced immunisation against HPV for girls in 2008, with the bivalent vaccine (Cervarix®). The vaccine offered was changed to the quadrivalent vaccine in 2012 following a UK‐wide review by the UK Joint Committee for Vaccines and Immunisation (JCVI) and subsequently to the nonavalent vaccine in 2022 The dosage schedule was reduced from three to two in 2015 and to one in 2022. Vaccination for gay, bisexual and other men who have sex with men (GBMSM) was introduced in 2017 and gender–neutral vaccination followed in 2019. Screening began at age 20, with first invitations issued age 19·5 years, until June 2016, at which time the age for entry into the screening programme was raised to 25 years.

Scotland's cervical screening IT system (Scottish Cervical Call‐Recall System SCCRS) contains a woman's entire screening record, including histology of cervical specimens and HPV immunisation status. Scotland can therefore monitor the impact of the HPV immunisation programme at both a population and an individual level, and has demonstrated the effectiveness of immunisation through a comprehensive programme of surveillance that monitors vaccine uptake, HPV prevalence, herd immunity, cervical disease, colposcopy performance, performance of screening tests, the uptake of cervical screening, and the influence of confounding factors.^4^, ^7^, ^8^, ^9^, ^10^, ^11^, ^12^, ^13^ We have recently reported changes in HPV distribution in high grade CIN and also reductions in invasive cancer.^14^, ^15^ We now report vaccine efficacy against CIN2+ and CIN3+ over at least four and a half years in women, born between January 1988 and June 1996, who have been received the bivalent vaccine and who have attended for screening, using population data derived from the cytology‐based screening programme rather than data from surveillance sampling.

## 2 METHODS

### 2.1 Design and participants

This population‐based retrospective national evaluation of the impact of HPV vaccination used routinely collected data on all women in Scotland born on or after 1 January 1988 up to 6 June 2016 inclusive who were eligible for the national cervical cancer screening programme. The cohort for evaluation includes women who were not eligible for HPV vaccination (birth years 1988–1990), women offered vaccination as part of a catch‐up programme during 2008 and 2009, given at ages 14–18 years (years of birth 1991–1994), and the routine programme from 2008 onwards, administered at ages 12–13 (years of birth 1995 and 1996). All immunised women received the bivalent vaccine. In the screened population, 3131 individuals were vaccinated at age greater than 18. Of these, 865 were vaccinated after eligibility for screening at age 20. To ensure accurate vaccination status assignation, these individuals (0.3% of the screened population) are excluded from the analysis. All analyses were conducted using only women attending for screening at least once in the period of observation, as CIN is almost exclusively a disease detected by cervical screening. CIN as an incidental finding is uncommon. A three dose schedule was in place for the cohort being studied. Three vaccination statuses have been used: unvaccinated (no vaccine received), incomplete (one dose or two doses 1 month apart) and complete (two doses at least 5 months apart or three doses).

### 2.2 Data sources, linkage and governance

The study analysed data collected as part of routine NHS Scotland clinical activity and recorded in SCCRS. Demographic data, screening events and cytology results, all histology results, HPV vaccination history (date and number of doses received), and postcode of residence were obtained from SCCRS, censored at 1 August 2020. Histological diagnosis (as a SNOMED code) for all cervical high grade CIN was obtained from SCCRS. The postcode was used to derive the Scottish Index of Multiple Deprivation (SIMD), an area‐based measure of deprivation that considers income, employment, education, health, access to services, crime and housing, and ranks from most (SIMD1) to least (SIMD5) deprived. The final dataset was anonymised before analysis.

Appropriate information governance protocols, detailed in Palmer et al., 2024, were followed.^15^

### 2.3 Statistical analysis

Estimated rates of CIN2+ and CIN3+ were stratified by the vaccination status described above, SIMD quintile, age vaccinated (routine, catch‐up and unvaccinated cohort) and the combination of age vaccinated and number of doses. Individuals were followed up until either disease was recorded or the follow up period ended (August 2020) whichever was the sooner.

Estimates of vaccine effectiveness against CIN2+ and CIN3+ were estimated from Cox Proportional Hazards models (CoxPH) to account for the variation in the follow‐up period(s) available for different individuals and changes in risk of CIN over time. CIN risk also changes with age and this is accounted for in two ways—adjusting for age using a categorical temporal age variable in the dose‐specific analysis and stratification of vaccine effectiveness results by age at vaccination (corresponding to year of birth cohort). VE was calculated as 100*(1‐HR) where HR is the hazard ratio (HR) and 95% confidence interval (CI) of the relevant vaccinated group compared to the unvaccinated population, estimated from the CoxPH model. The reference group for analyses by vaccination status comprised unvaccinated women from all age cohorts. Kaplan–Meier curves were created to show the accrual of disease over time in different groups. Herd protection was investigated by comparing the disease rates among unvaccinated women in the 1991–1992, 1993–1994, and 1995–1996 cohorts (who were eligible for catch‐up or routine vaccination) with unvaccinated women in the 1988–1990 cohort.

Statistical analysis was conducted in R version 4.1.2. Disease rates and CoxPH models were calculated using the survival package. The Kaplan–Meier curves were created using the survminer package. A *p*‐value ≤0·05 was considered significant.

## 3 RESULTS

### 3.1 Baseline characteristics

A total of 271,896 individual records of women who attended for screening along with their screening results were obtained from SCCRS. Restricting the analysis to women who had attended for screening removed 48 cases of invasive carcinoma (out of 239) in women who had not attended for screening but no cases of high grade CIN. The cases of invasive cancer not included in this analysis were retained in the analysis of vaccination effect on invasive carcinoma (Palmer et al.).^15^ 3610 women (1.3% of the total cohort) were screened before the age of 20, with the majority of these (2287) screened when aged over 19. As there were 395 cases of high grade CIN in this group, it has been retained within the cohort for analysis. Table 1 shows the cohort broken down by year of birth, deprivation, and vaccination status.

**TABLE 1:** Demographics of the SCCRS cohort stratified by vaccine status.

		Screening attendance at least one screen	Vaccine status^a^					
			Unvaccinated		Incomplete		Complete	
		*n*	*n*	(%)	*n*	(%)	*n*	(%)
Birth cohort	1988	39,068	39,068	100.00	0	0	0	0
	1989	36,920	36,875	99.9	29	0.1	16	0.04
	1990	36,707	31,068	86.1	1157	3.2	3942	10.7
	1991	35,214	15,081	42.8	3025	8.6	17,108	48.6
	1992	33,692	10,587	31.4	2456	7.3	20,649	61.3
	1993	30,505	9474	31.1	2304	7.6	18,727	61.4
	1994	27,468	5874	21.4	427	5.4	20,118	73.2
	1995	23,168	3031	13.1	429	1.8	19,710	85.1
	1996^b^	8289	925	11.2	117	1.4	7247	87.4
SIMD quintile^c^	1 (Most)	58,421	33,923	58.1	3328	5.7	21,170	36.2
	2	55,383	31,158	56.3	2560	4.6	21,665	39.1
	3	54,384	30,688	56.4	2038	3.7	21,658	39.8
	4	46,590	25,149	54.0	1553	3.3	19,888	42.7
	5 (Least)	48,160	27,242	56.6	1166	2.4	19,752	41.0
	Unknown	8093	4363	53.9	346	4.3	3384	41.8

^a^ Vacciation status: Unvaccinated: no doses given; Incomplete: one dose or two doses 1 month apart; Complete: two doses at least 5 months apart or 3 doses.

^b^ The 1996 cohort were subject to a change in the cervical screening age. Those born until 5 June 1996 were eligible for screening at age 20 hence the 1996 year only represents 5/12 of all women born in 1996.

^c^ SIMD: Scottish index of Multiple Deprivation is derived from the postcode of residence.

### 3.2 Unadjusted analyses

**FIGURE 1:**
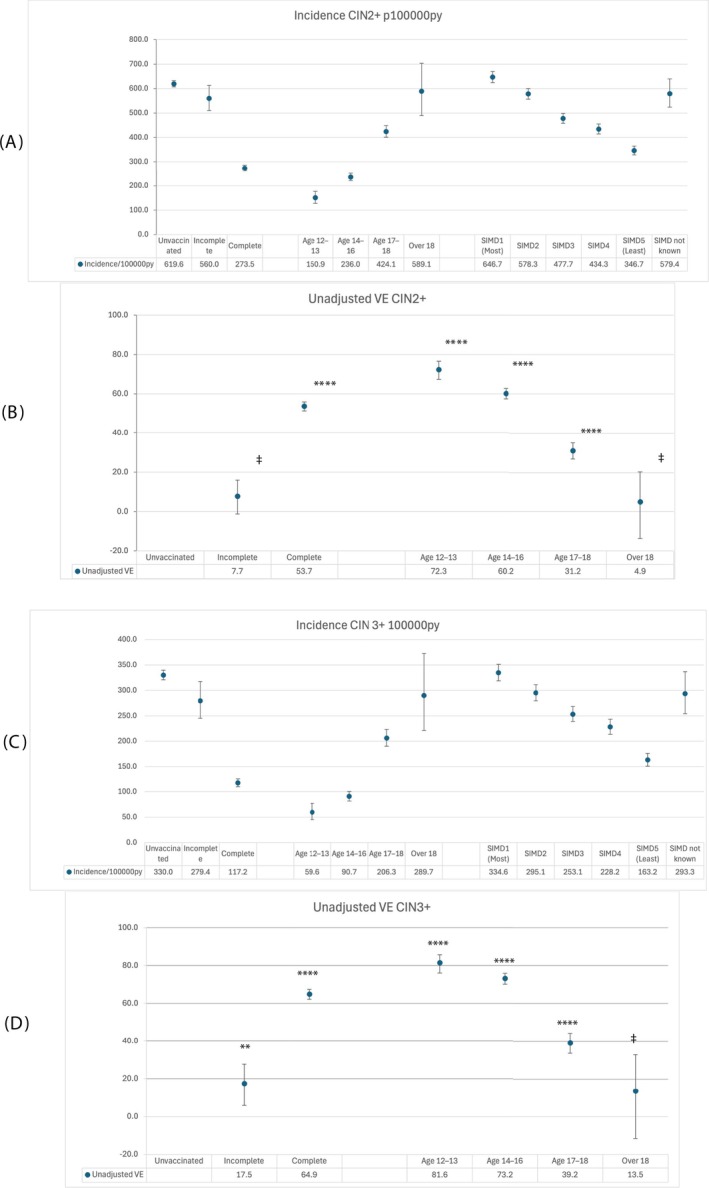
Estimates of CIN2+ and CIN3+ incidence per 100,000 py and unadjusted vaccine effectiveness by vaccine status, age at vaccination and Scottish Index of multiple deprivation, with 95% confidence intervals. *p*‐values denoted by ‡ (>0.05), * (0.05 ≤ *p* ≥.01), ** (0.01< *p* ≥0.001), *** (0.001< *p* ≥.0001), ****p <.0001). Reference group: Unvaccinated women. Panel (A) CIN2+ incidence; panel (B) CIN2+ vaccine effectiveness; panel C: CIN3+ incidence; panel D: CIN3+ vaccine effectiveness.

The results of unadjusted analysis of incidence rates per 100,000 person‐years (py) and vaccine effectiveness are presented in Figure 1 and supplementary Table 1. The supplementary table contains data on denominators, person‐years of follow up, cases of disease and CIs.

There are significant reductions in incidence of CIN2+ from 619.6 (95% CI: 607.0–632.5) to 273.5 (95% CI: 26.7–285.8) following complete vaccination at any age, corresponding to a VE of 53.7% (95%CI: 51.3–55.9) and for CIN3+, from 330.0 (95% CI: 320.8–339.4) to 117.2 (95% CI: 109.5–125.3) following complete vaccination at any age, corresponding to a VE of 64·9 (95% CI: 62.2–67.4). Incomplete vaccination at any age showed a weak but significant positive effect against CIN3+ (VE: 17.5 (95% CI: 5.9, 27.6)) but no significant effect against CIN2+. Taking account of an individual's changing age during the period of follow‐up gave reduced but still significant VE for full vaccination (CIN2+: 42.6 (39.7, 45.4), *p* <.0001; CIN3+ 54.2 (50.7, 57.5), *p*<.0001) and no significant VE for incomplete vaccination (CIN2+: −7.1 (−17.5–2.4) *p* = .148; CIN3+: 0.69 (−13.2–12.9), *p* = .917).

Significant reduction in incidence was observed with any vaccination at age 12/13 for CIN2+, from 619.6 (95% CI: 607.0–632.5) for all unvaccinated women to 150.9 (95% CI: 127.5–177.4); VE 72.3% (95%CI: 67.4–76.6), and at age 14–16, to 236.0 (95% CI: 221·3–251.5); VE 60·2% (95%CI: 57.4–62.8). Immunisation at age 17–18 showed a significant but smaller reduction in incidence and in VE (incidence: 424.1; 401.0–448.2; VE: 31.2; 27.0–25.1). Similar reductions were seen for CIN3+ (rate for all non‐immunised women: 330.0 (95% CI: 320.8–336.4); for age 12/13: 59.6 (95%CI: 45·2–77.0); VE 81·6% (95%CI: 76.1–85.8): for age 14–16: 90.7 (95% CI: 81.6–100.4); VE 73.2% (95%CI: 70.2–76.0); for age 17–18: 206·3 (95% CI:190.3–223.3); VE 39.2 (95% CI:33.8–44.1)). Vaccination over the age 18 showed no significant VE (*p* = .26).

Incidence is lower in deprivation strata SIMD 2–5 with respect to the individuals from the most deprived areas (SIMD 1) for both CIN2+ and CIN 3+. For CIN2+, incidence fell from 646.7 (95% CI 624.4–669.7; SIMD 1) to 578.3 (95% CI 556.7–600.6; SIMD 2), 477.7 (95% CI 457.9–498.0; SIMD 3), 434.3 (95% CI 414.0–455.4; SIMD 4) and 346.7 (95% CI 328.9–365.2; SIMD 5). The incidence for women for whom the SIMD quintile was not known was similar to that for SIMD 2. A similar trend was observed for CIN 3+. The incidence for women from the most deprived areas (SIMD 1) was 334.6 (95% CI: 318·6–351.2) compared to 295.1 (95% CI: 279.7–311.1; SIMD 2), 253.1 (95% CI: 238.8–268.1; SIMD 3), 228.2 (95% CI: 213.5–243.6; SIMD 4), and 163.2 (95% CI: 151.1–176.1; SIMD 5). Again, the incidence in women for whom the SIMD quintile was not known was similar to SIMD 2.

### 3.3 Adjusted analyses

**FIGURE 2:**
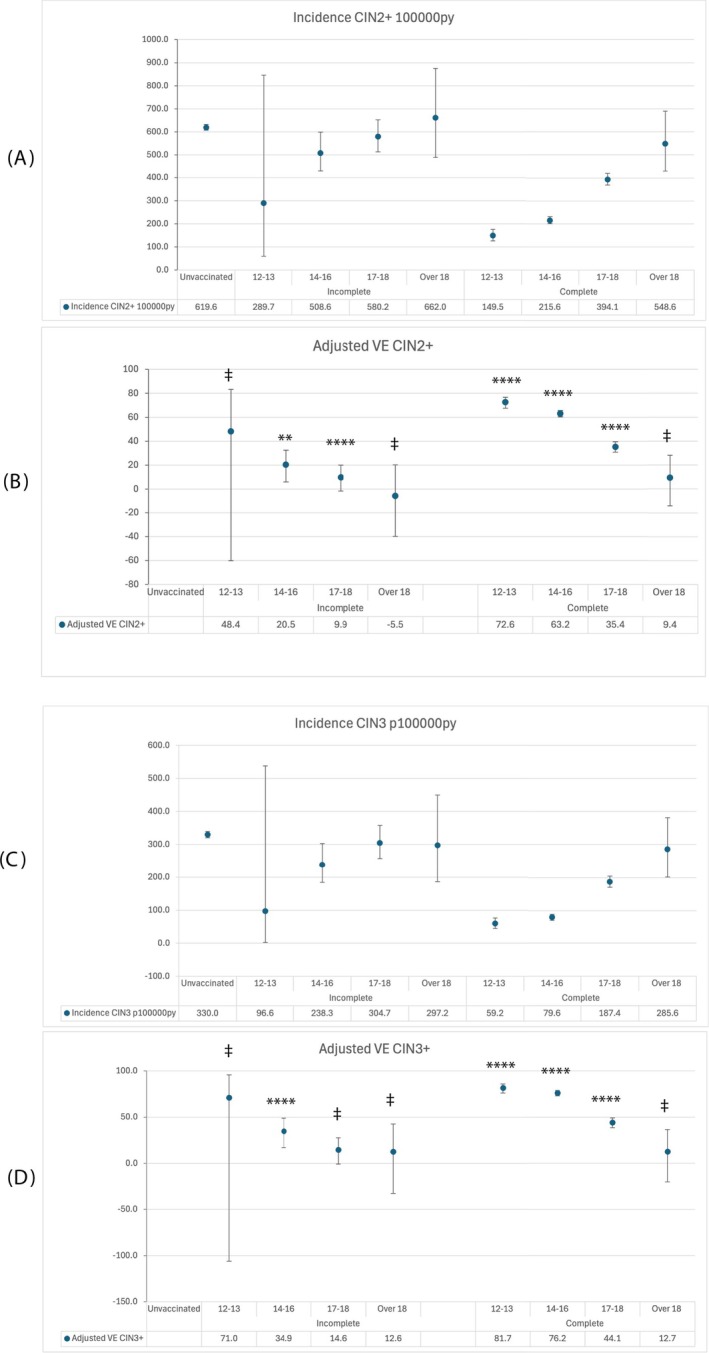
Estimates of CIN2+ and CIN3+ incidence per 100,000 py and adjusted vaccine effectiveness by the combined factor of vaccine status and age at vaccination, with 95% CIs. *p*‐values denoted by ‡ (>0.05), * (0.05 ≤ *p* ≥.01), ** (0.01< *p* ≥.001), *** (0.001< *p* ≥.0001), ****p <.0001). Reference group: Unvaccinated women. Panel (A) CIN2+ incidence; panel (B) CIN2+ vaccine effectiveness; panel (C) CIN3+ incidence; panel (D) CIN3+ vaccine effectiveness.

**FIGURE 3:**
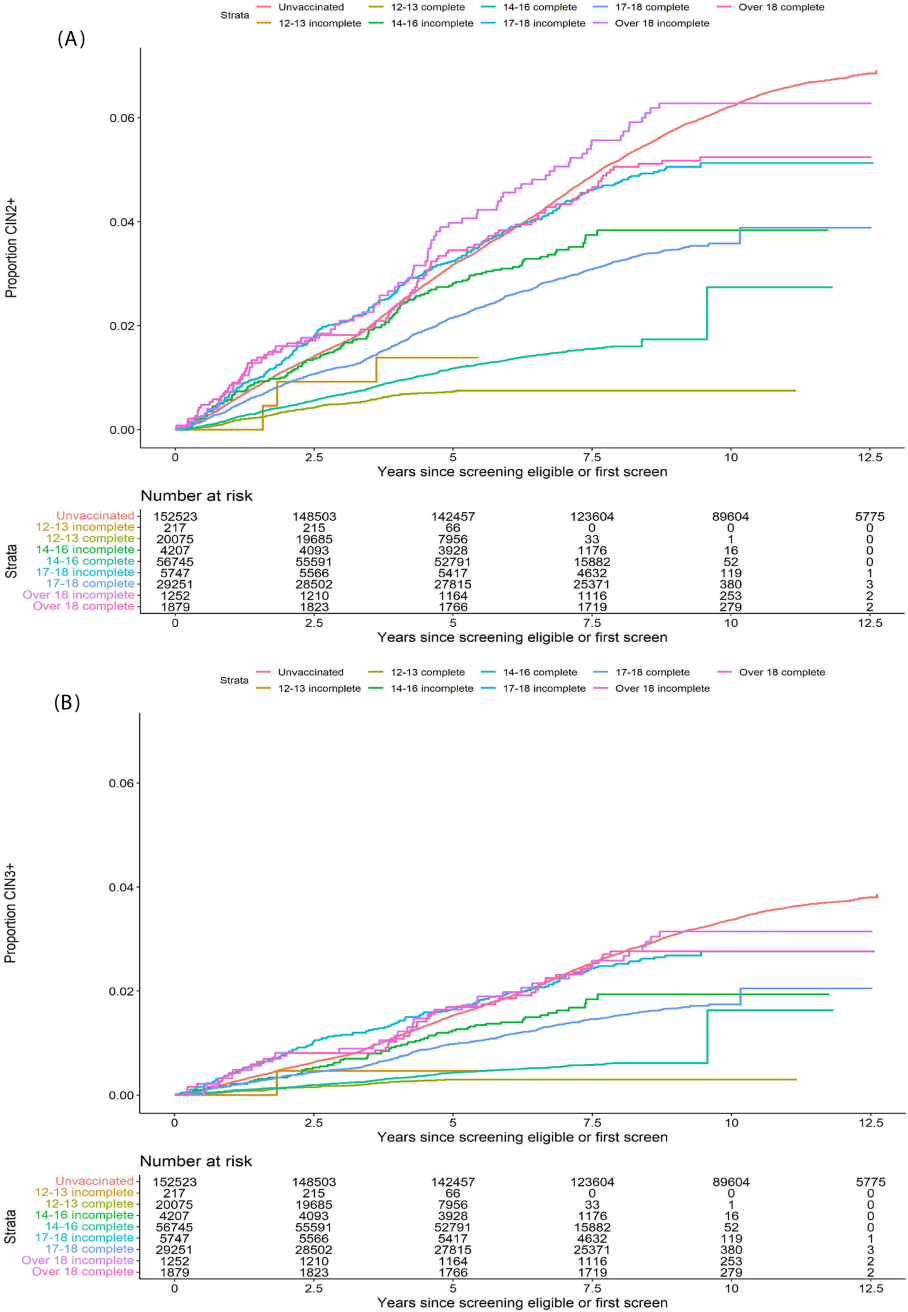
Proportion of screened population with CIN2+ (panel A) and CIN3+ (panel B) over time. Cases censored at first diagnosis of disease or end of follow‐up period, whichever is the sooner.

The analysis by age at vaccination stratified by vaccination status, following adjustment by deprivation, is shown in Figure 2 and in supplementary Table 2. Kaplan–Meier plots show accrual of disease over time since eligibility for screening (age 20) or first screen, which ever was the earlier, by age and vaccination status (Figure 3). The supplementary table contains data on denominators, person‐years of follow up, cases of disease and CIs.

Reductions in incidence of both CIN2+ and CIN3+ are seen following complete vaccination at ages 12 and 13, at age 14–16 and at age 17–18, compared to no vaccination.

For CIN2+ and complete immunisation, the incidence following vaccination at age 12–13 was 149.5 (CI: 126.0–176.0), and at age 14–16,215·6 (CI: 201.0–231.0), compared to unvaccinated: 619.3 (CI: 607.0–632.5), giving VE of 72·6 (67.7–76·8, *p* <.0001) and 63.2 (CI 60.4–65.8; *p* <.0001), respectively. Vaccination at age 17–18 years was also associated with significant reduction in incidence (349.1/100,000 [CI: 369.9–419.6]) and VE of 35.4 (CI: 30.9–39.5, *p* <.0001) when compared to the unvaccinated, but vaccination over the age of 18 was not associated with any significant VE.

For CIN3+ and complete immunisation, incidence following vaccination at age 12–13 years was 59.2 (CI: 44.8–76.6), at 14–16 years 79·6 (CI: 70.8–89.2), and at 17–18 years 187·4 (CI: 170.8–205.2) compared to 330.0 (CI: [320.8–339.4]) for unvaccinated individuals, giving VE of 81.7 (CI [76.2–85.9], *p* <.0001), 76·2(CI [73.2–78.9] *p* <.0001) and 29.0 (CI (23·0–34.6, *p* <.0001)), respectively.

Despite large positive estimates for VE of incomplete immunisation at age 12–13, the CIs are wide due to the small number of women in this group. Adjusted VE following incomplete immunisation at age14 was 20.5 (95%CI: 6.1–32.6) for CIN2+ and 34.9 (95%CI:17.0–48.9) for CIN3+.

No significant benefit was seen following any vaccination over the age of 18 years at either endpoint.

### 3.4 Deprivation

**FIGURE 4:**
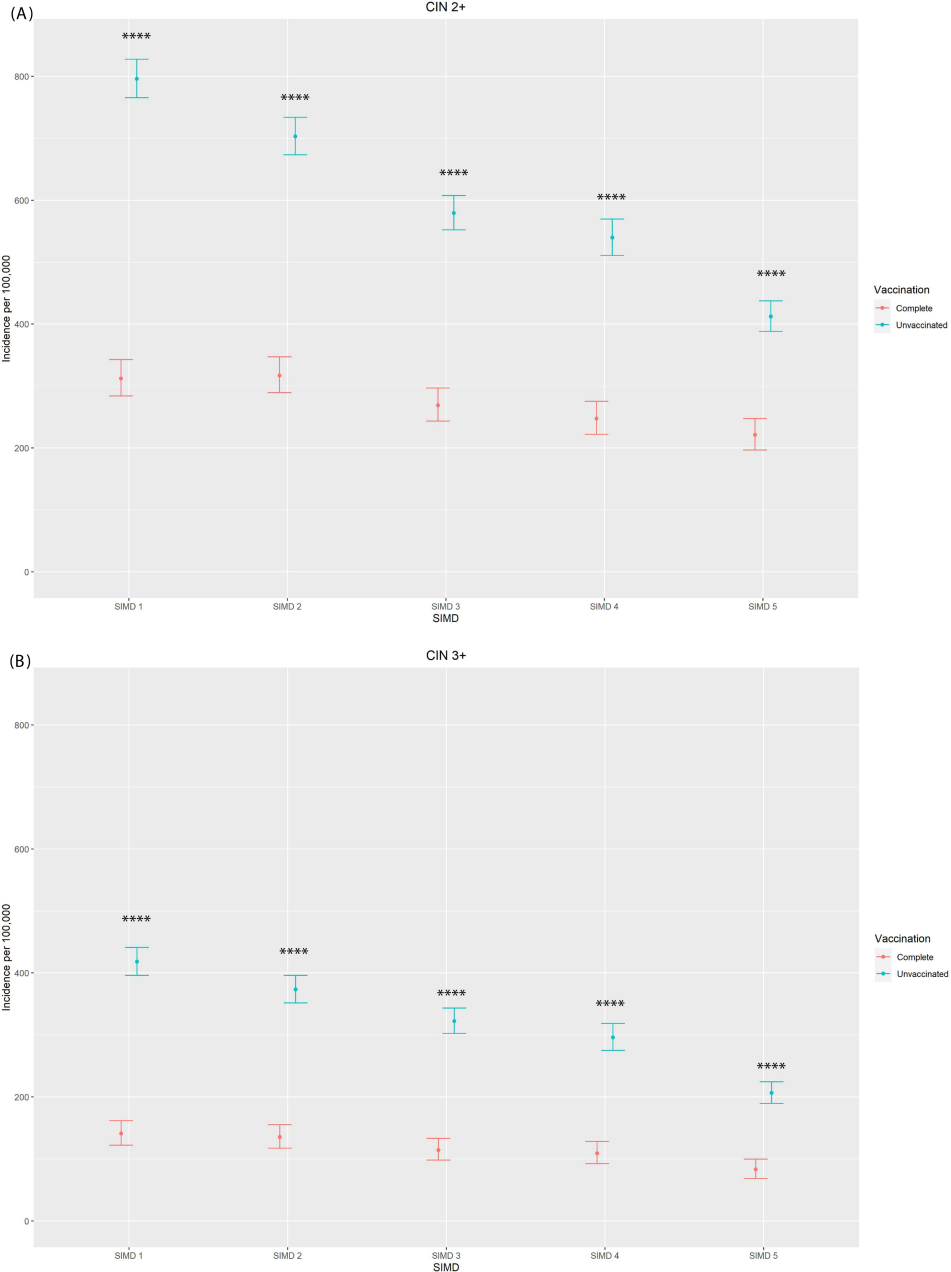
Incidence of CIN2+ (panel A) and CIN3+ (panel B) per 100,000 py with 95% CI by vaccination status (complete or unvaccinated) and deprivation quintile (SIMD1—most deprived; SIMD5—least deprived). *p*‐values denoted by ‡ (>0.05), * (0.05≤ *p* ≥.01), ** (0.01< *p* ≥.001), *** (0.001< *p* ≥.0001), ****p <.0001). Reference group: Unvaccinated women.

Stratification of incidence by SIMD and vaccination status showed reductions in incidence of both CIN2+ and CIN3+ in all quintiles, with the largest reduction for women residing in the most deprived areas (SIMD1) and the smallest reduction for women residing in the least deprived areas (SIMD5). Significant positive VE is demonstrated in every quintile, in both CIN2+ and CIN3+, again with higher VE in the more deprived quintiles The differences between quintiles is greater for CIN2+ than for CIN3+ (Figure 4). Supplementary Table 3 contains data on vaccine effectiveness and CI.

### 3.5 Herd protection

Herd protection was assessed by comparing incidence rates in the unvaccinated women in each year of birth stratum with the incidence rates in unvaccinated women not eligible for vaccination (years of birth 1988–1990). Herd protection is seen in all year of birth strata eligible for vaccination. The 1991–1992 and 1993–1994 strata (age at vaccination 17 and 16, and 15 and 14, respectively) represent the catch‐up cohort, and the 1995–1996 stratum (age at vaccination 13 and 12 years) represents the routine cohort. For both CIN2+ and CIN3+ the HRs decline with younger age of vaccination suggesting a greater level of herd protection in individuals vaccinated earlier in adolescence. (Table 2).

**TABLE 2:** Incidence of CIN2+ and CIN3+ and hazard ratios in unvaccinated women by year of birth cohort.

CIN grade/cohort (year of birth)	Denominator	CIN2+							CIN3+						
		Cases of disease	Person years follow up	Incidence rate per 100,000py	Incidence rate 95% CI	HR	HR 95% CI	*p*‐value	Cases of disease	Person years follow up	Incidence rate per 100,000py	Incidence rate 95% CI	HR	HR 95% CI	*p*‐value
1988–1990	107,551	7569	1,132,669	668.2	(653.3, 683.5)				4017	1,132,669	354.6	(248·6, 264.5)			
1991–1992	25,668	1094	217,468.8	503.1	(473.7, 533.8)	0.75	(0.70, 0.80)	<.0001	617	217,468.8	283.7	(134.3, 157.5)	0.76	(0.70, 0.83)	<.0001
1993–1994	15,348	422	101,765.5	414.7	(376.1, 456.2)	0.63	(0.57, 0.70)	<.0001	212	101,765.5	208.3	(71.9, 94.4)	0.55	(0.48, 0.63)	<.0001
1995–1996	3956	33	19,583.85	168.5	(116.0, 266.6)	0.28	(0.20, 0.39)	<.0001	10	19,583.85	51.1	(6.2, 23.9)	0.14	(0.08, 0.26)	<.0001

*Note*: The denominator is the number of unvaccinated women in each year of birth stratum.

## 4 DISCUSSION

In their review of long‐term effectiveness data from trials and real‐world studies of HPV vaccination, Kurosawa et al. (2022) examined data from both clinical trials and real world studies of HPV vaccination but found no population‐based reports of the long‐term effectiveness of the bivalent vaccine.^6^ We present the first linked population‐based data on the long‐term clinical effectiveness of the bivalent HPV vaccine in preventing high grade CIN. Our analysis uses data on 271,031 women with at least one screen born between 1 January 1988 and 6 June 1996. A total of 152,523 women were unvaccinated, 10,991 had only one dose or two doses 1 month apart, and 107,517 had received three doses or two doses at least 5 months apart. The majority of the vaccinated women received the vaccine between 12 and 18 years, and only a small number were vaccinated over the age of 18 years. The follow‐up period from administration of the vaccine is at least 12 years, and from date of first screen is between 4.5 and 12 years from the first cervical screen. Only women vaccinated before being eligible for screening have been included in the analysis.

The adjusted vaccine effectiveness of three doses, or two doses at least 5 months apart, of the bivalent vaccine, given at age 12 or 13 years was 72·6 (95% CI 67·7–76·8) against CIN2+, and 81·7(95% CI: [76·2–85·9]) against CIN3+. Although these estimates are lower than those reported at 12 months following first screen at age 20 for the same cohort of women (VE for CIN2+: 89 (95% CI:81–94); CIN3+: 86, (95% CI 75–92)), this reduction was anticipated due to the longer follow‐up over subsequent screening rounds allowing more time for disease to develop. The confidence limits of the CIN3+ estimates overlap, but those for CIN2+ do not.^4^ Nevertheless, the findings indicate strong continued vaccine protection consistent with trial data from both the Patricia and Costa Rica trials, which demonstrated good clinical effectiveness and persistence of antibody levels for up to 14 years post vaccination.^5^

In addition to the longer follow‐up, the decline in VE for CIN2+ observed over time with our cohort may be related to the unmasking of infections caused by non‐vaccine HPV types due to the absence of HPV 16 and 18, as suggested by Shing et al. (2022), who also reported declining VE in immunised women in the Costa Rica trial when followed up to 11 years post vaccination.^3^ The decline in VE would also be in keeping with another anticipated effect of the vaccine—the change in HPV distribution in high grade CIN, particularly CIN2, which has been confirmed following both quadrivalent and bivalent vaccination.^14^, ^16^, ^17^ Following vaccination with the bivalent vaccine, the relative proportion of CIN2 associated with non‐vaccine related HPV has increased substantially, with a smaller increase seen in CIN3.^14^ Infections by the non‐vaccine protected and cross‐protected types may have a slower progression to high grade CIN, resulting in presentation and detection at an older age.^18^ Finally, the peak age of incidence of CIN3+ in Scotland is in the mid‐to‐late 20s. Nevertheless, the small reduction in VE does not negate the very considerable benefit of vaccination over no vaccination in the prevention of high grade pre‐invasive cervical disease.

The real‐world population‐based study of the quadrivalent vaccine most comparable to this study is from Denmark (Thamsborg et al., 2020), which reported 10‐year observations on women born in 1993, immunised at age 15 and screened from age 23.^19^ They reported relative risk with respect to a cohort born in 1983, who were not offered vaccination and were followed for the same length of time. The relative risk of CIN2+ was 0.74 (95% CI 0.66–0.82) and for CIN3+ 0.68 (95% CI 0.58–0.79), corresponding to VE of 26% and 32% respectively. These results are somewhat lower than those observed for the most comparable Scottish cohort (age 14–16 years). However, the Scottish 14–16 cohort in this study included younger girls less likely to be sexually active before vaccination, possibly explaining the difference in effectiveness.

The data also show that herd protection has been maintained over the follow‐up period. Indeed, when comparing the results of this analysis to a similar one performed on data from disease at first screen (Palmer et al., 2019), the herd effect appears to have strengthened over time, now extending to encompass cohorts immunised aged 14 and over.^4^ This may reflect the increasing proportion of routinely immunised women in the sexually active population over the last 10 years, which will have reduced HPV prevalence and transmission within the community. This will have fed back through sexual mixing to older cohorts offered vaccine as part of the catch up programme, in which vaccine uptake was lower. In the future, vaccinated boys will most probably strengthen the magnitude of herd protection, but routinely vaccinated boys will probably not have influenced the present analysis as they are unlikely to have been sexually active in significant numbers at the time the data was extracted (2020).

Whilst it is possible to measure herd protection by combining information on vaccine effect, vaccine uptake and HPV type distribution in CIN to estimate the expected numbers of cases of CIN prevented in the vaccinated group and comparing this estimate with the overall numbers of cases in the cohorts eligible for vaccination, this is complicated in the case of HPV‐related disease by the variation in HPV type distribution in the different grades of CIN, the variation in time since vaccination within the immunised population, and the change in background rates of CIN over time and with age. Single‐cohort vaccination programmes would be more amenable to this approach than the current multi‐cohort study population. Comparing the disease in the unvaccinated women in the vaccine‐eligible cohorts with unvaccinated women in cohorts not eligible for vaccination will give a more precise and robust indication of herd protection in multi‐cohort populations.

The higher incidence of cervical disease in more deprived communities is well recognised. The data presented show very clearly that the relationship between deprivation and cervical disease incidence has been attenuated considerably in completely vaccinated women. The effect of HPV vaccination is considerably greater in women from the most deprived areas than in women from the least deprived areas. It is known that women who have been vaccinated have a better uptake of cervical screening than unvaccinated women, and this may have amplified the effect of the vaccine, leading to the stronger overall effect of vaccination in more deprived groups, who are known to be less likely to participate in screening.^9^, ^20^ In addition, as the more deprived women are less likely to be vaccinated, the strong herd protection demonstrated by this data may also be contributing to the reduction in disease incidence in these groups.^13^

For both CIN2+ and CIN3+, the data do not show any benefit from vaccination for women over the age of 18 or for those with incomplete vaccination and who had their first dose at the ages of 17 or 18. However, significant vaccine effectiveness has now been demonstrated for incomplete vaccination between the ages of 14 and 16. Although a high degree of VE is shown for incomplete vaccination at age 12–13, the small numbers in this particular group give wide CIs. Complete vaccination at age 18 or younger confers significant benefit, the magnitude of the benefit increasing with younger age at vaccination. The Kaplan Meier curves showing the accrual of disease over time demonstrate that all the strata have crossed the unvaccinated curve, including those vaccinated over the age of 18 years. Further observation may show a benefit in groups not yet showing significant VE, although this may be related to herd protection rather than direct vaccine protection.

These data continue the observations on one and two doses in Palmer et al. (2019) and in Palmer et al. (2024), assessing protection against CIN2+ and CIN3+ at first screen and against invasive cancer respectively.^4^, ^15^ The findings support the implementation of one‐dose schedules, the evidence for which has been summarised by the UK Joint Committee on Vaccines and Immunisation (JCVI).^21^ The lack of significant benefit of incomplete vaccination in the routine cohort (vaccinated at age 12–13 years), most probably due to the small number of women in this group, is counter‐balanced by the significant protective effect of incomplete vaccination demonstrated in young adolescents, aged 14–16 at vaccination.

This study has several strengths. It uses directly linked immunisation, screening, pathology and demographic data gathered from a national cervical screening programme that has been curated to remove duplicate records. Scotland has maintained a robust HPV immunisation surveillance system since 2008 and there is confidence in the completeness and reliability of the data. As a result, the findings are relevant to real‐life screening and cancer elimination programmes. Comparable results were observed from the principal analysis, which included screened women only, and a sensitivity analysis that accounted for age at diagnosis of disease. As CIN is a disease almost always only diagnosed in women attending for screening, there is no certain knowledge of CIN prevalence in unscreened women. Therefore, an analysis based on screened women provides estimates of VE that are less open to bias. Disease rates are generally reported as age standardised rates, based on population rather than screened individuals, which may result in lower estimates compared to those reported here.^22^ Disease rates in screened and unscreened women may eventually converge due to the development of strong herd protection as the spectrum of HPV types in screened and unscreened women was not significantly different when analysed in Scotland before vaccination started.^23^

The principal shortcoming of this analysis is its inability to provide robust estimates of the VE of fewer than three doses, or two doses given at least 5 months apart, of vaccination at age 12–13, but this is due to the success of the Scottish HPV immunisation programme in achieving very high levels of uptake of the recommended doses. In addition, as it is a study based on aggregated screening data, it is not possible to comment upon the factors underlying the small decline in vaccine efficacy. However, data for HPV prevalence from the English surveillance programme, derived from residual material from the English Chlamydia screening programme, shows that the reductions in HPV16/18, reported by Kavanagh et al. and mirrored in England, have been maintained.^7^, ^24^ HPV 16/18 prevalence was 0·6% in women aged 16–18 in 2020. Women born between 2002 and 2004 would have received 2 doses of the quadrivalent vaccine at age 12–13 years. HPV 31, 33, and 45 also declined in English cohorts vaccinated with the bivalent vaccine and the reduction in these types has been maintained in those receiving the quadrivalent vaccine. Presently, no screening‐generated comprehensive HPV typing data is available, and so the next phase of the Scottish surveillance programme has been designed to gather data on HPV prevalence in the community, and HPV types present in HPV—screen positive samples, in biopsies containing CIN, and in invasive cancers. This will enable assessment of, inter alia, the consequences of moving to the quadrivalent vaccine with its lesser degree of cross‐protection against HPV 31, 33, and 45.

The results reinforce the importance of continued positive messaging about the value of HPV immunisation. They also indicate that, as the immunised cohorts move through screening programmes, it will be necessary to review the organisation and management of such programmes and clinical management protocols to avoid the harms that necessarily follow from screening. HPV immunisation has been shown to reduce the performance of cytology screening tests, and also of HPV screening tests.^10^, ^11^ Colposcopy performance has also been shown to be affected by the reduction in prevalence of high grade CIN.^12^, ^13^ The change in distribution of HPV genotypes, with the near‐elimination of HPV 16 and 18 in routinely vaccinated populations, may also affect the overall progression of HPV infection to CIN as well as the rate at which CIN3 and invasive cancer develop. As the impact and biology of non‐vaccine protected types becomes clearer, it may be necessary to revise the HPV spectrum detected by screening tests, as has been suggested by Kann.^16^ Furthermore, it will be essential for studies of the relationship between HPV immunisation and disease to include CIN3+ and not just CIN2+, the current default. In time, with evidence‐supported changes in screening and management protocols, reporting of CIN2+ may no longer be informative. Communication of evidence‐based changes to screening and management will need to emphasise the positive aspects to avoid the impression of removal of services or benefit. Changes in HPV type distribution both in the population and in cervical disease will also inform the development and implementation of new vaccines and vaccination protocols.

In summary, HPV vaccination remains the best way of preventing the personal, social and economic burden of HPV‐related cancers world‐wide, given the expense of current organised screening programmes. Nevertheless, where screening programmes exist, they will need to be continued for some time to come. The use of routine screening data gathered from age 20 and the long duration of the surveillance programme means that this study is able to present individual‐derived clinical data over a similar period of time to the randomised trials. The population benefit of HPV vaccination appears to be long‐lasting, with clinical benefit being seen at least 12 years after vaccination at age 12–13 years. High uptake of vaccine confers additional benefits of widening herd protection and reduction in the adverse effects of deprivation.

## AUTHOR CONTRIBUTIONS

**Timothy J. Palmer:** Conceptualization; data curation; writing – original draft; writing – review and editing. **Kimberley Kavanagh:** Formal analysis; methodology; writing – original draft; writing – review and editing. **Kate Cuschieri:** Writing – original draft; writing – review and editing; conceptualization. **Ross L. Cameron:** Writing – review and editing; data curation. **Catriona Graham:** Data curation; writing – review and editing. **Allan Wilson:** Writing – review and editing. **Kirsty Roy:** Writing – review and editing; supervision.

## FUNDING INFORMATION

The Scottish Government, through core funding of Public Health Scotland. The funding source played no part in the design, execution, analysis and interpretation, or preparation of the manuscript for publication.

## CONFLICT OF INTEREST STATEMENT

TP, KK, RC, CG, AW and KR report no conflicts of interest. KC reports the following: My institution has received R&D funding (or gratis consumables to support R&D) in the last 3 years from the following: Abbott, Euroimmun, GeneFirst, Qiagen, Hiantis, SeeGene, Roche, HOLOGIC, Barinthus Biotech and Daye. I have attended advisory board meetings for HOLOGIC (UK travel paid) and Abbott (UK travel paid) and have received support for conference attendance from COPAN.

## Supporting information


Supplementary Tables S1–S3:
**Supplementary Table 1:** Estimates of CIN2+ and CIN3+ incidence and unadjusted vaccine effectiveness by vaccine status, age at vaccination and Scottish Index of Multiple Deprivation.
**Supplementary Table 2:** Estimates of CIN2+ and CIN3+ incidence and adjusted vaccine effectiveness by the combined factor of vaccine status and age at vaccination.
**Supplementary Table 3:** Incidence of CIN2+ and CIN3+ and vaccine efficacy in unvaccinated and completely vaccinated women by deprivation cohort.

## Data Availability

Access to data is available on request from Public Health Scotland upon completing the appropriate documentation. This is available from phs.statsgov@phs.scot and is required for audit purposes. Other information is available from the corresponding author on request’.

## References

[1] www.who.int/cancer/prevention/diagnosis-screening/cervical-cancer/en/

[2] Susanne KK , Mari N , Karin S , et al. Final analysis of a 14‐year long‐term follow‐up study of effectiveness and immunogenicity of the quadrivalent human papillomavirus vaccine in women from four Nordic countries. eClinicalMedicine. 2020;23:100401. 10.1016/j.eclinm.2020.100401 32637895 PMC7329692

[3] Shing JZ , Hu S , Herrero R , et al. Precancerous cervical lesions caused by non‐vaccine‐preventable HPV types after vaccination with the bivalent AS04‐adjuvanted HPV vaccine: an analysis of the long‐term follow‐up study from the randomised Costa Rica HPV vaccine trial. Lancet Oncol. 2022;23(7):940‐949. 35709811 10.1016/S1470-2045(22)00291-1 PMC9255557

[4] Palmer T , Wallace L , Pollock K , et al. Prevalence of cervical disease at age 20 after immunisation with bivalent HPV vaccine at age 12‐13 in Scotland: retrospective population study. BMJ. 2019;365:l1161. 30944092 10.1136/bmj.l1161 PMC6446188

[5] Drolet M , Bénard É , Pérez N , Brisson M , HPV Vaccination Impact Study Group . Population‐level impact and herd effects following the introduction of human papillomavirus vaccination programmes: updated systemic review and meta‐analysis. Lancet. 2019;394:497‐509. doi:10.1016/S0140-6736(19)30298-3 31255301 PMC7316527

[6] Kurosawa M , Sekine M , Yamaguchi M , et al. Long‐term effect of human papillomavirus vaccination in clinical trials and real‐world data—a systematic review. Vaccine. 2022;10:256. doi:10.3390/vaccines10020256 PMC8877934 35214713

[7] Kavanagh K , Pollock KG , Cuschieri K , et al. Changes in the prevalence of human papillomavirus following a national bivalent human papillomavirus vaccination programme in Scotland: a 7‐year cross‐sectional study. Lancet Infect Dis. 2017;17(12):1293‐1302. 28965955 10.1016/S1473-3099(17)30468-1

[8] Cameron RL , Kavanagh K , Pan J , et al. Human papillomavirus prevalence and herd immunity after introduction of vaccination program, Scotland, 2009–2013. Emerg Infect Dis. 2016;22(1):56‐64. doi:10.3201/eid2201.150736 26692336 PMC4696690

[9] Palmer TJ , McFadden M , Pollock KGJ , et al. HPV immunisation and increased uptake of cervical screening in Scottish women; observational study of routinely collected national data. Br J Cancer. 2016;114:576‐581. doi:10.1038/bjc.2015.473 26794278 PMC4782202

[10] Palmer TJ , McFadden M , Pollock KGJ , et al. HPV immunisation and cervical screening—confirmation of changed performance of cytology as a screening test in immunised women: a retrospective population‐based cohort study. Br J Cancer. 2016;114:582‐589. doi:10.1038/bjc.2015.474 26931370 PMC4782203

[11] Bhatia R , Kavanagh K , Cubie HA , et al. Use of HPV testing for cervical screening in vaccinated women—insights from the SHEVa (Scottish HPV prevalence in vaccinated women) study. Int J Cancer. 2016;138:2922‐2931. doi:10.1002/ijc.30030 26845632

[12] Munro A , Gillespie C , Cotton S , et al. The impact of HPV type on colposcopy performance in women offered HPV immunisation in a catch‐up vaccine programme: a two centre observational study. BJOG. 2017;124:1394‐1401. doi:10.1111/1471-0528.14563 28102931

[13] Cameron RL , Kavanagh K , Watt DC , et al. The impact of bivalent HPV vaccine on cervical intraepithelial neoplasia by deprivation in Scotland: reducing the gap. J Epidemiol Community Health. 2017;71:954‐960. 28756395 10.1136/jech-2017-209113

[14] Cuschieri K , Palmer T , Graham C , Cameron R , Roy K . The changing nature of HPV associated with high grade cervical lesions in vaccinated populations, a retrospective study of over 1700 cases in Scotland. Br J Cancer. 2023;129(7):1134‐1141. doi:10.1038/s41416-023-02386-9 37563221 PMC10539290

[15] Palmer T , Kavanagh K , Cuschieri K , et al. Invasive cervical cancer incidence following bivalent HPV vaccination—a population‐based observational study of age at immunisation, dose, and deprivation. JNCI. 2024;116(6):857‐865. doi:10.1093/jnci/djad263 38247547

[16] Kann H , Hortlund M , Eklund C , Dillner J , Faust H . Human papillomavirus types in cervical dysplasia among young HPV‐vaccinated women: population‐based nested case‐control study. Int J Cancer. 2020;146(9):2539‐2546. 31868230 10.1002/ijc.32848

[17] Pimenoff VN , Gray P , Louvanto K , Soderlund‐Strand A , Dillner J , Lehtinen M . Ecological diversity profiles of non‐vaccine‐targeted HPVs after gender‐based community vaccination efforts. Cell Host Microbe. 2023;31:1921‐1929. doi:10.1016/j.chom.2023.10.001 37944494

[18] Powell N , Cuschieri K , Cubie H , et al. Alison Fiander cervical cancers associated with human papillomavirus types 16, 18 and 45 are diagnosed in younger women than cancers associated with other types: a cross‐sectional observational study in Wales and Scotland (UK). J Clin Virol. 2013;58(3):571‐574. doi:10.1016/j.jcv.2013.08.020 24051043

[19] Thamsborg L , Napolitano G , Larsen L , Lynge E . High‐grade cervical lesions after vaccination against human papillomavirus: a Danish cohort study. Acta Obstet Gynecol Scand. 2020;99:1290‐1296. 32754966 10.1111/aogs.13935 PMC7540379

[20] https://publichealthscotland.scot/media/19729/2023-05-30-cervical-screening-report.pdf accessed 15 February 2024

[21] JCVI statement on a one‐dose schedule for the routine HPV immunisation. https://www.gov.uk/government/publications/single‐dose‐of‐hpv‐vaccine‐jcvi‐concluding‐advice/jcvi‐statement‐on‐a‐one‐dose‐schedule‐for‐the‐routine‐hpv‐immunisation‐programme accessed 31/05/2023.

[22] https://www.opendata.nhs.scot/dataset/annual‐cancer‐incidence/resource/72c852b8‐ee28‐4fd8‐84a9‐5f415f4bc325 accessed 1 January 2025

[23] Kavanagh K , Sinka K , Cuschieri K , et al. Estimation of HPV prevalence in young women in Scotland; monitoring of future vaccine impact. BMC Infect Dis. 2013;13:519 http://www.biomedcentral.com/1471-2334/13/519 24188790 10.1186/1471-2334-13-519 PMC4228358

[24] Checchi M , Mesher D , Panwar K , Anderson A , Beddows S , Soldan K . The impact of over ten years of HPV vaccination in England: surveillance of type‐specific HPV in young sexually active females. Vaccine. 2023;41:6734‐6744. doi:10.1016/j.vaccine.2023.10.002 37821315

