# Supplementary material for: Sustained impact of bivalent HPV immunisation on CIN incidence over two rounds of cervical screening
Source: Int J Cancer. 2025 Nov 3;158(5):1348–60. doi: 10.1002/ijc.70183 (PMC12765965; doi:10.1002/ijc.70183)
Supplement: Supplementary file 1 — Supplementary Tables S1–S3: Supplementary Table 1: Estimates of CIN2+ and CIN3+ incidence and unadjusted vaccine effectiveness by vaccine status, age at vaccination and Scottish Index of Multiple Deprivation. Supplementary Table 2: Estimates of CIN2+ and CIN3+ incidence and adjusted vaccine effectiveness by the combined factor of vaccine status and age at vaccination. Supplementary Table 3: Incidence of CIN2+ and CIN3+ and vaccine efficacy in unvaccinated and completely vaccinated women by deprivation cohort. [file IJC-158-1348-s001.pdf]

Sustained impact of bivalent HPV immunisation on CIN incidence over two rounds of cervical screening

Timothy J Palmer, Kimberley Kavanagh, Kate Cuschieri, Ross L Cameron, Catriona Graham, Allan Wilson, Kirsty Roy

Contents

Supplementary Table 1	Estimates of CIN2+ and CIN3+ incidence and unadjusted vaccine effectiveness by vaccine status, age at vaccination and Scottish Index of Multiple Deprivation.
Supplementary Table 2	Estimates of CIN2+ and CIN3+ incidence and adjusted vaccine effectiveness by the combined factor of vaccine status and age at vaccination
Supplementary Table 3	Incidence of CIN2+ and CIN3+ and vaccine efficacy in unvaccinated and completely vaccinated women by deprivation cohort

Supplementary Table 1

				CIN2+						CIN3+					
		Denominator	Person years follow-up	Cases of disease	Incidence rate per 100,000py	Incidence rate 95% CI	Unadjusted VE	Unadjusted VE 95% CI	p-value	Cases of disease	Incidence rate per 100,000 py	Incidence rate 95% CI	Unadjusted VE	Unadjusted VE 95% CI	p-value
Vaccine status [1]	Un-vaccinated	152523	1471487	9118	619.6	(607.0; 632.5)	Reference	..	..	4856	330.0	(320.8; 339.4)	Reference	..	..
	Incomplete	10991	84471.47	473	560.0	(510.6, 612.8)	7.7	(-1.2; 15.9)	0.089	236	279.4	(244.9, 317.4)	17.5	(5.9, 27.6)	0.0042
	Complete	107517	734440.3	2009	273.5	(261.7; 285.8)	53.7	(51.3; 55.9)	<0.0001	861	117.2	(109.5,125.3)	64.9	(62.2, 67.4)	<0.0001
Age at vaccination	Un-vaccinated	152523	1471487	9118	619.6	(607.0; 632.5)	Reference	..	..	4856	330.0	(320.8; 339.4)	Reference	..	..
	12-13	20292	97385.22	147	150.9	(127.5; 177.4)	72.3	(67.4; 76.5)	<0.0001	58	59.6	(45.2;77.0)	81.6	(76.1; 85.8)	<0.0001
	14-18	60952	403743.8	953	236.0	(221.3; 251.5)	60.2	(57.4; 62.8)	<0.0001	366	90.7	(81.6; 100.4)	73.2	(70.2; 76.0)	<0.0001
	17-18	34998	297073.6	1260	424.1	(401.1; 448.2)	31.2	(27.0; 35.2)	<0.0001	613	206.3	(190.3; 223.3)	39.2	(33.8; 44.1)	<0.0001
	Over 18	2266	20709.18	122	589.1	(489.2, 703.4)	4.9	(-13.7; 20.5)	0.58	60	289.7	(221.1, 372.9)	13.5	(-11.6,33.0)	0.26
SIMD quintile [2]	1 (Most)	58421	489846.5	3168	646.7	(624.4, 669.7)	..	..	..	1639	334.6	(318.6, 351.2)	..	..	..
	2	55383	466001.2	2695	578.3	(556.7, 600.6)	..	..	..	1375	295.1	(279.7, 311.1)	..	..	..
	3	54384	461400.9	2204	477.7	(457.9, 498.0)	..	..	..	1168	253.1	(238.8, 268.1)	..	..	..
	4	46590	394384.9	1713	434.3	(414.0, 455.4)	..	..	..	900	228.2	(213.5, 243.6)	..	..	..
	5 (Least)	48160	409903.5	1421	346.7	(328.9, 365.2)	..	..	..	669	163.2	(151.1, 176.1)	..	..	..
	Not known	8093	68861.87	399	579.4	(524.0, 639.2)	..	..	..	202	293.3	(254.3, 336.7)	..	..	..

Estimates of CIN2+ and CIN3+ incidence and unadjusted vaccine effectiveness by vaccine status, age at vaccination and Scottish Index of multiple deprivation.

Footnote:

1 Vaccination status: Unvaccinated: no doses given; Incomplete: one dose or two doses one month apart; Complete: two doses at least 5 months apart or 3 doses.

2 SIMD: Scottish index of Multiple Deprivation is derived from the postcode of residence.

Supplementary Table 2

Combined vaccination status and age at vaccination (years) [1]	Denominator	Person years follow-up	CIN2+						CIN3+					
			Cases of disease	Incidence rate per 100,000py	Incidence rate 95% CI	Adjusted VE [2]	Adjusted VE [2] 95% CI	p-value	Cases of disease	Incidence rate per 100,000py	Incidence rate 95% CI	Adjusted VE [2]	Adjusted VE [2] 95% CI	p-value
Unvaccinated	152523	1471487	9118	619.6	(607.0, 632.5)				4856	330	(320.8, 339.4)			
12-13 Incomplete	217	-	-	289.7	(59.8, 846.7)	48.4	(-60.2,83.3)	0.253	-	96.6	(2.4, 538.1)	71	(-105.9, 95.9)	0.216
12-13 Complete	20075	96349.8	144	149.5	(126.0, 176.0)	72.6	(67.7,76.8)	<0.0001	57	59.2	(44.8, 76.6)	81.7	(76.2, 85.9)	<0.0001
14-16 Incomplete	4207	28116.8	143	508.6	(428.7, 599.1)	20.5	(6.1,32.6)	0.007	67	238.3	(184.7, 302.6)	34.9	(17.0, 48.9)	0.001
14-16 Complete	56745	375627	810	215.6	(201.0, 231.0)	63.2	(60.4,65.8)	<0.0001	299	79.6	(70.8, 89.2)	76.2	(73.2, 78.9)	<0.0001
17-18 Incomplete	5747	47917.4	278	580.2	(514.0, 652.5)	9.8	(-1.6,20.0)	0.09	146	304.7	(257.3, 358.3)	14.6	(-0.8, 27.6)	0.062
17-18 Complete	29251	249156.2	982	394.1	(369.9, 419.6)	35.4	(30.9,39.5)	<0.0001	467	187.4	(170.8, 205.2)	44.1	(38.5, 49.2)	<0.0001
Over 18 Incomplete	820	7401.9	49	662.0	(489.7, 875.2)	-5.5	(-39.8,20.3)	0.56	22	297.2	(186.3, 450.0)	12.6	(-32.8, 42.5)	0.527
Over 18 Complete	1446	13307.3	73	548.6	(430.0, 689.7)	9.4	(-14.1,28.0)	0.367	38	285.6	(202.1, 391.9)	12.7	(-20.2, 36.5)	0.405

Estimates of CIN2+ and CIN3+ incidence and adjusted vaccine effectiveness by the combined factor of vaccine status and age at vaccination

Footnote.

1 Vaccination status: Unvaccinated: no doses given; Incomplete: one dose or two doses one month apart; Complete: two doses at least 5 months apart or 3 doses.

2 adjusted by SIMD

Supplementary Table 3

Disease grade and deprivation quintile [1]	CIN2+							CIN3+						
	Incidence rate per 100,000py	Incidence rate 95% CI	Incidence rate per 100,000py	Incidence rate 95% CI	VE	VE 95% CI	p value	Incidence rate per 100,000py	Incidence rate 95% CI	Incidence rate per 100,000py	Incidence rate 95% CI	VE	VE 95% CI	p value
	Unvaccinated [2]		Complete [2]					Unvaccinated [2]		Complete [2]				
SIMD1	796.0	(765·5, 827·4)	312.1	(283·7, 342·4)	59.0	(54·6, 63·0)	<0.0001	418.0	(396·0, 441·0)	141.6	(122·8, 162·6)	66.6	(61·2, 71·3)	<0.0001
SIMD2	703.1	(673·4, 733·8)	313.9	(285·9, 343·9)	53.1	(48.0, 57·7)	<0.0001	373.3	(351·7, 395·8)	132.5	(114·6, 152·5)	64.6	(58·7, 69·7)	<0.0001
SIMD3	579.2	(552·2, 607·3)	266.4	(240·8, 294·0)	51.5	(45·7, 56·6)	<0.0001	322.3	(302·2, 343·4)	113.7	(97·2, 132·2)	64.5	(58·0, 69·9)	<0.0001
SIMD4	539.6	(510·9, 569·4)	246.7	(221·1, 274·5)	51.4	(45·0, 57.0)	<0.0001	296.1	(274·9, 318·4)	107.6	(90·9, 126·5)	64.8	(57·8, 70·6)	<0.0001
SIMD5	412.2	(388·1, 437·4)	220.5	(196·72, 246·9)	44.8	(37·0, 51·6)	<0.0001	206.3	(189·4, 224·3)	83.0	(68·0, 99·4)	61.3	(52·4, 68·5)	<0.0001

Incidence of CIN2+ and CIN3+ and vaccine efficacy in unvaccinated and completely vaccinated women by deprivation cohort.

Footnote:

1. SIMD: Scottish Index of Multiple Deprivation, where SIMD1 is the most deprived and SIMD5 is the least deprived.

2. Unvaccinated: no vaccine given; Complete vaccination: two doses at least 5 months apart or 3 doses.
